# Adalimumab clinical efficacy is associated with rheumatoid factor and anti-cyclic citrullinated peptide antibody titer reduction: a one-year prospective study

**DOI:** 10.1186/ar1851

**Published:** 2005-11-09

**Authors:** Fabiola Atzeni, Piercarlo Sarzi-Puttini, Donata Dell' Acqua, Simona de Portu, Germana Cecchini, Carola Cruini, Mario Carrabba, Pier Luigi Meroni

**Affiliations:** 1 Rheumatology Unit, Department of Medicine, L Sacco University Hospital, 74 Via GB Grassi, 20157 Milano, Italy; 2 CIRF/Center of Pharmacoeconomics, Faculty of Pharmacy, University of Naples, Federico II, Napoli, Italy; 3 Allergy, Clinical Immunology and Rheumatology Unit, Department of Internal Medicine, University of Milan, IRCCS Istituto Auxologico Italiano, Milano, Italy

## Abstract

Studies on autoantibody production in patients treated with tumor necrosis factor-α (TNF-α) inhibitors reported contradictory results. We investigated in a prospective study the efficacy of a treatment with human monoclonal anti-TNF-α antibody (adalimumab) in patients with rheumatoid arthritis (RA) and we evaluated the relationship between treatment efficacy and the incidence and titers of disease-associated and non-organ-specific autoantibodies. Fifty-seven patients with RA not responsive to methotrexate and treated with adalimumab were enrolled. Antinuclear, anti-double-stranded(ds)DNA, anti-extractable nuclear antigens, anti-cardiolipin (aCL), anti-β_2 _glycoprotein I (anti-β_2_GPI) autoantibodies, rheumatoid factor (RF) and anti-cyclic citrullinated peptide (anti-CCP) autoantibodies were investigated at baseline and after 6 and 12 months of follow-up. Comparable parameters were evaluated in a further 55 patients treated with methotrexate only. Treatment with adalimumab induced a significant decrease in RF and anti-CCP serum levels, and the decrease in antibody titers correlated with the clinical response to the therapy. A significant induction of antinuclear autoantibodies (ANA) and IgG/IgM anti-dsDNA autoantibodies were also found in 28% and 14.6% patients, respectively, whereas aCL and anti-β_2_GPI autoantibodies were not detected in significant quantities. No association between ANA, anti-dsDNA, aCL and anti-β_2_GPI autoantibodies and clinical manifestations was found. Clinical efficacy of adalimumab is associated with the decrease in RF and anti-CCP serum levels that was detected after 24 weeks and remained stable until the 48th week of treatment. Antinuclear and anti-dsDNA autoantibodies, but not anti-phospholipid autoantibodies, can be induced by adalimumab but to a lower extent than in studies with other anti-TNF blocking agents.

## Introduction

Clinical trials in rheumatoid arthritis (RA) have demonstrated that tumor necrosis factor-α (TNF-α) blocking agents are highly beneficial for most patients refractory to classic treatment with disease-modifying anti-rheumatic drugs [1-4]. However, a significant proportion of patients are still relatively resistant to such a therapy [5]. No reliable markers predictive for the clinical response have been identified, although a recent report suggests that a decrease in rheumatoid factor (RF) and anti-cyclic citrullinated peptide (anti-CCP) antibody titers might be a useful adjunct in assessing the efficacy of treatment [6]. A decrease in IgM-RF titers was initially described by Charles and colleagues in a small series of patients receiving infliximab [7], but then inconsistent findings were reported [8-11].

Recently, two papers showed a decrease in RF and anti-CCP antibody titers in patients with RA treated with infliximab [6,8]. In both studies the decrease paralleled the improvement in disease activity score, but one group reported a return to baseline titer levels by prolonging the follow-up to 54 and 78 weeks [8]. In contrast, autoantibodies against non-organ-specific autoantigens have been reported during treatment with TNF-α blocking agents. Thus, antinuclear (ANA) and anti-double-stranded DNA (anti-dsDNA) autoantibodies have been respectively described in up to 86% and 57% of patients with RA treated with the TNF-α blocking agent infliximab [3,7,12-16]. Lower percentages were reported in patients treated with etanercept [17]. Interestingly, these autoantibodies were only anecdotally associated with clinical manifestations suggestive of a drug-induced systemic lupus erythematosus [17]. As regards anti-dsDNA autoantibodies, the occurrence of low-affinity autoantibodies of the IgM or IgA isotype was thought to explain the lack of such an association, in contrast with the widely accepted relationship between high-affinity anti-dsDNA IgG autoantibodies and systemic lupus erythematosus [13]. Although ANA and anti-dsDNA autoantibodies have been reported at higher prevalence in patients treated with infliximab than in those treated with etanercept and in spite of the lack of any flare in a patient with previous infliximab-induced systemic lupus erythematosus when etanercept therapy was started, the occurrence of these autoantibodies has been considered a drug class-related side effect [17,18].

Finally, anti-phospholipid autoantibodies – detectable mainly by the anti-cardiolipin (aCL) assay – were also reported in patients with RA receiving TNF-α blockers. In some cases their appearance was related to concomitant infectious processes [19], but again contrasting results were reported and no correlation with the clinical manifestations specific for the anti-phospholipid syndrome was clearly found [8,9,16]. However, a paper suggested that they might be predictive of a poor clinical outcome [20].

Adalimumab, a fully human anti-TNF-α monoclonal antibody, was recently approved for the treatment of both moderate and severe RA [4,21,22]. The present 1-year study was planned to evaluate the following in a prospective manner: first, the clinical efficacy of adalimumab; second, whether the prevalence and titers of RA-associated autoantibodies such as RF and anti-CCP autoantibodies correlate with treatment effect; and third, whether non-organ-specific autoantibodies are induced by adalimumab as reported for other TNF-α blocking agents.

## Materials and methods

### Patient sera

Fifty-seven patients (53 women and 4 men; mean age at baseline 56 years (range 28 to 83)) with refractory RA were included in the study. The patients were selected in accordance with the inclusion criteria of Adalimumab Research in Active RA (ReAct), an open-label multicenter, multinational phase IIIb study conducted primarily in Europe. In the ReAct study, patients were assigned to receive single self-injections of adalimumab subcutaneously at 40 mg every other week in addition to their pre-existing but inadequate therapies [22]. All patients fulfilled the 1987 American College of Rheumatology (ACR) classification criteria for RA [23] and were treated with methotrexate (mean dosage 10 mg per week (range 7.5 to 20)) and adalimumab (40 mg every other week as a single dose by subcutaneous injection). In addition 55 patients with RA treated with methotrexate only were followed up and evaluated with comparable parameters at 6-month intervals.

Written informed consent was obtained from all patients and the study was approved by the Research and Ethics Committee of the L Sacco University Hospital in Milan.

Demographic and clinical data are presented in Table 1. During the study, 42 patients of the adalimumab group received concomitant corticosteroids (7.5 mg per day), 48 received non-steroidal anti-inflammatory drugs and/or analgesics, and 6 received other drugs. Patients were followed clinically at regular intervals by the same physician during this period and in particular when they were receiving adalimumab. Clinical assessment included the number of tender and swollen joints, the duration of morning stiffness, erythrocyte sedimentation rate (ESR), and C-reactive protein (CRP) (Table 2). Clinical features suggestive of infections or autoimmune disorders were also recorded. ESR and CRP and significant concomitant clinical features suggestive of infections or autoimmune disorders were recorded accurately (Table 2). The DAS 28 criteria [24,25] were applied to assess clinical efficacy. Eighteen patients discontinued adalimumab treatment before the end of the study, between 3 and 12 months, because of adverse events, treatment inefficacy or severe infectious disease.

Blood was drawn between 08:00 and 09:00 in the morning when the patients visited the outpatient clinic on day 0 (screening evaluation), and after 6 and 12 months of treatment. The blood was immediately centrifuged and the serum was stored at -80°C.

### Detection of RF and anti-CCP autoantibodies

Tests for IgM RF and anti-CCP autoantibodies were performed at baseline and after 6 and 12 months of adalimumab treatment. IgM RF was measured by immunonephelometry with the quantitative N Latex RF system (Dade Behring, Marburg, Germany). RF titers higher than 15 IU/ml were considered positive. Anti-CCP autoantibodies were tested by using a second-generation commercially available ELISA kit (Menarini Diagnostics, Florence, Italy) as described [26]. In brief, 100 μl of anti-CCP standards (0, 2, 8, 30 and 100 U/ml), and samples from controls and patients (diluted 1:100 in PBS) were distributed into individual wells. The microtiter plates were coated with highly purified synthetic cyclic peptides containing modified arginine residues. After incubation for 60 minutes, the wells were washed three times with 200 μl wash buffer (borate buffer containing 0.8% sodium azide).

The microplates were then incubated for 30 minutes at room temperature (22–24°C) with alkaline-phosphate-labelled murine monoclonal antibody against human IgG and washed again three times. A chromogenic substrate solution (Mg^2+ ^phenolphthalein monophosphate buffered solution) was added to each well. After 30 minutes the reaction was stopped with sodium hydroxide-EDTA-carbonate buffer. The absorbance was read at 550 nm. Serum samples were evaluated in triplicate, and the upper normal limit (15 arbitrary units (AU)/ml) was assumed in accordance with the manufacturer's recommendations. To follow the changes in antibody levels during therapy, all serum samples displaying a high concentration (more than 100 AU/ml) were evaluated after a further 1:10 dilution and then corrected for this additional dilution factor. To avoid any plate-to-plate variation of anti-CCP measurements, plates from the same batch (batch number 470094) were used; the inter-assay and intra-assay variabilities were less than 9%.

### Detection of ANA

Anti-nuclear autoantibodies were performed at baseline and after 6 and 12 months of adalimumab treatment, by indirect immunofluorescence with HEp2 cells as described [27]. Titers of more than 1:160 were considered positive. Sera positive for ANA by indirect immunofluorescence were further analysed for the presence of anti-extractable nuclear antigens (anti-ENA) by Addressable Laser Bead Immunoassays (Menarini Diagnostics) in accordance with the manufacturer's instructions [28].

Tests for anti-dsDNA IgG autoantibodies were performed at baseline and after 6 and 12 months of treatment with adalimumab by using enzyme-immunoassay (EIA) (Pharmacia Diagnostics, Friburg, Germany); positive samples were also evaluated by Farr assay and by indirect immunofluorescence with *Crithidia luciliae *(CLIFT) as described [29]. Anti-dsDNA autoantibodies of the IgM isotype have been also detected by CLIFT with a specific anti-human μ chain antiserum (MP Biomedicals, Aurora, OH, USA).

### Detection of anti-phospholipid autoantibodies

Anti-phospholipid autoantibodies (aPL) were detected as aCL and as anti-β_2 _glycoprotein I (anti-β_2_GPI) by ELISA as described [30]; values were expressed as IgG or IgM aPL units (GPL or MPL, respectively; considered positive when more than 10 GPL or MPL units) for aCL and as OD values for anti-β_2_GPI autoantibodies (results higher than the 95th percentile of 50 normal healthy controls were considered positive: more than 0.130 for IgG and more than 0.280 for IgM) [30]. Anti-cardiolipin and anti-β_2_GPI autoantibodies were evaluated at baseline and after 6 and 12 months of adalimumab treatment. The sera of the control group of patients with RA were analyzed twice with a 1-year interval.

### Statistics

Statistical analysis (95% and 99% confidence intervals) was performed with the χ^2 ^test when applicable and with Fisher's exact test in other conditions (when the expected value under null hypothesis was less than 5). Wilcoxon's test was applied in comparisons of continuous variables. Correlations were expressed with Spearman's rank correlation coefficient. Probability (*p*) values less than 0.05 were considered statistically significant. Data were analyzed with SPSS statistical software 10.00 for Windows (SPSS, Inc, Chicago, IL, USA). Statistical analysis was calculated by Last Observation Carried Forward (LOCF).

## Results

### Response to therapy

An ACR 20 response was achieved by 88% of patients at 24 weeks, and by 80% at 48 weeks. ACR 50 (ACR 70) percentages were 51 (54) and 14 (26) at 24 and 48 weeks, respectively.

Table 2 reports the decrease in DAS 28 values, the tender and swollen joint counts and the ESR and CRP values during the study. The group of patients treated with methotrexate alone displayed a stable disease activity during the study with no significant changes in all the clinical assessment parameters (data not shown).

### Modification of anti-CCP antibody and RF titers during adalimumab treatment

At baseline, 46 of the 57 patients with RA (80.7%) were positive for anti-CCP autoantibodies, and 43 of 57 (75%) were positive for RF. A strong correlation between anti-CCP and RF at baseline was observed (χ^2^, *p *< 0.001). Although no patients who were positive for anti-CCP or RF at baseline became negative after anti-TNF-α treatment, both RF serum titers and anti-CCP autoantibodies decreased significantly at week 24 (*p *< 0.01 and *p *< 0.01, respectively) and week 48 (*p *< 0.01 and *p *< 0.01, respectively) (Table 2). When we grouped the patients on the basis of their clinical response (ACR improvement criteria) to adalimumab, a significant decrease in RF serum titers was observed in those who were clinically improved according to ACR 20 and ACR 50 at weeks 24 and 48, whereas a decrease was also found for ACR 70 at week 48 (Table 3). Anti-CCP antibody titers declined in patients who were clinically improved according to ACR 50 at week 24 and in those who were improved according to ACR 20, ACR 50 and ACR 70 at week 48 (Table 4). The Spearman's correlation coefficient of the improvement in the DAS 28 values with the decrease in RF titer at week 48 was 0.316 (*p *= 0.018), and that for the anti-CCP autoantibodies titer was 0.33 (*p *= 0.013). No significant change in RF and anti-CCP titers was observed in patients treated with methotrexate alone (data not shown).

### Occurrence of ANA in patients treated with adalimumab

At baseline, 4 of 57 (7%) adalimumab-treated patients with RA, and 5 of 55 (9%) methotrexate-treated patients with RA tested positive for ANA (Table 5). After 12 months of therapy, induction of ANA was observed in 16 of 57 (28%) adalimumab-treated patients with RA, and in 8 of 55 (14.5%) with methotrexate only (Table 5). The difference in ANA positivity before and after the end of follow-up was statistically significant for the adalimumab-treated group only (*p *< 0.01). All the induced ANAs were still positive at the end of the study, including 6 of 18 patients who discontinued the treatment.

No patient was positive for IgG or IgM anti-dsDNA autoantibodies in either the adalimumab-treated group or the methotrexate-treated group at baseline. By solid-phase ELISA, the presence of IgG anti-dsDNA autoantibodies was observed in 2 of 57 (3.5%) adalimumab-treated patients with RA after 6 months of treatment and in 4 of 57 (7%) after 12 months of treatment (Table 5). All the positive samples displayed low antibody titers (less than 25 AU/ml, normal value 17 AU/ml, mean values +5 SD for 100 normal blood donors, matched for age and sex). The positive samples displayed low titers (less than 1/80) in the CLIFT assay and were negative in the Farr assay (data not shown). Immunoglobulin G anti-dsDNA autoantibodies were associated with positivity for ANA, and remained positive until the end of the study, including three of four positive patients who discontinued the treatment.

Five of 57 (5.1%) and 7 of 57 (14.6%) patients receiving adalimumab were positive for IgM anti-dsDNA autoantibodies after 24 and 48 weeks of treatment, respectively. The occurrence of IgM anti-dsDNA autoantibodies was associated with the presence of IgG anti-dsDNA only in two patients at the 24th week and in four patients at the 48th week after treatment. None of the patients with RA treated only with methotrexate displayed IgG or IgM anti-dsDNA autoantibodies during follow-up. As regards anti-ENA autoantibodies, three patients were positive for anti-Ro (52 and 60 kDa) before adalimumab and two additional patients became positive during treatment. Four patients were positive for anti-Ro (52 and 60 kDa) autoantibodies before methotrexate treatment and a further one became positive for anti-Ro at the end of follow-up (Table 5).

The presence of anti-nuclear and anti-dsDNA autoantibodies was not associated with any clinical event.

### Occurrence of anti-phospholipid autoantibodies in patients with RA treated with adalimumab

No patients with RA were positive for aCL or for anti-β_2_GPI autoantibodies at baseline. At the end of the study, aCL were detected at low titers (less than 20 GPL or MPL units) in two patients in both groups; anti-β_2_GPI autoantibodies were found in one patient in the adalimumab-treated group at low titers (IgG 0.201 and IgM 0.312 OD values, respectively) and in none of the patients treated with methotrexate only. All aCL and anti-β_2_GPI autoantibodies were detected in patients positive for ANA. No correlation was found between aPL and clinical status (including lupus-like symptoms or thrombosis) or the occurrence of side effects (including infections).

## Discussion

Anti-TNF-α agents, such as infliximab and etanercept, have been reported to be beneficial for patients with RA not responsive to conventional treatment [1-3,5]. Our study confirms that adalimumab, a new fully human anti-TNF-α monoclonal antibody, is also effective in improving the clinical scores in patients with RA. A decrease in RF and anti-CCP antibody titers has been recently correlated with clinical improvement after infliximab therapy in patients with RA [6-8]. It has been suggested that blocking TNF-α might have an inhibitory effect on the production of autoantibodies closely related to RA disease activity [6]. Actually, besides their diagnostic value, high RF serum levels were shown to be an independent predictor for deteriorating radiological damage, and a greater prevalence of anti-CCP autoantibodies was found in patients who develop severe radiological damage [31-36]. However, whereas RF titer reduction was also reported by other groups after infliximab and etanercept therapy [7,8,10,11,37-39], contrasting results were found for anti-CCP antibody levels [7,8,10,11,37-39]. Such a discrepancy might be related, at least in part, to the different periods of follow-up and to the methods used to measure the antibody levels in the different studies. In fact, most of the enrolled patients displayed an aggressive form of the disease with high anti-CCP antibody titers; however, not all the studies performed serial serum sample dilutions or tried to reduce the batch-to-batch variability in performing the solid-phase assays for anti-CCP detection. This could have made the laboratory tests too insensitive to detect variations in the antibody titers during treatment.

Our results show a significant decrease in anti-CCP autoantibodies and RF titers after both 6 and 12 months of adalimumab therapy. Thus, serial evaluation of these autoantibodies seems to be useful in monitoring the clinical course of patients with RA undergoing therapy with adalimumab, as previously suggested for infliximab [6]. It is possible that anti-CCP-positive patients with RA might display a more active disease associated with a higher response to therapy in comparison with patients negative for anti-CCP autoantibodies. This finding might explain, at least in part, the association between the clinical response and the decrease in anti-CCP titer. An additional study with a larger series of anti-CCP-negative patients with RA would be necessary to evaluate such a hypothesis, because the number of anti-CCP-negative patients in the present study was too small.

In contrast, results of studies on the association between the response to conventional RA treatment and the decrease in RF and/or anti-CCP autoantibodies have been inconsistent. A decrease in serum RF levels has been reported in association with successful treatment with methotrexate and gold salts [40]. A more recent study confirmed the association between decrease in RF titer and treatment response; in contrast, a shorter disease duration but not a specific treatment was associated with a decline in anti-CCP levels [39]. We did not find any variation in RF and anti-CCP antibody serum levels in the group of patients with RA who were treated with methotrexate alone. However, it must be pointed out that these patients did not display the same clinical characteristics as the patients with RA selected for the adalimumab treatment. Moreover, they were already receiving treatment with methotrexate when included and displayed a stable clinical course of the disease. It is therefore likely that a possible decrease in antibody titer could have already taken place, with no additional effect of treatment being detectable.

The mechanisms by which TNF-α blocking agents could lead to a decrease in titers of autoantibodies closely related to RA is still matter of speculation. Either the downregulation of pro-inflammatory processes and/or the modulation of apoptosis has been suggested to have a role in autoantibody production or in protein citrullination that eventually might trigger the B cell responses [17,41,42]. In contrast, we confirmed the development of ANA and anti-dsDNA autoantibodies in our adalimumab-treated patients. Although the prevalence of IgG anti-dsDNA was comparable to that recently reported by Keystone and Haraoui [43], the number of ANA positive patients was slightly larger at the end of the treatment. However, both autoantibodies occurred at lower frequency than those reported in series of patients treated with infliximab or etanercept [17].

As previously reported in patients treated with different anti-TNF-α blocking agents, an increased frequency of anti-dsDNA autoantibodies of the IgM isotypes have also been found in our patients treated with adalimubab [7]. Clinical monitoring of the subgroup of patients positive for ANA and anti-dsDNA autoantibodies did not show any manifestation potentially related to a full-blown lupus disease. Accordingly, IgG anti-dsDNA autoantibodies seemed to be at low titers and to display low affinity, as demonstrated by their negativity in the Farr assay. We did not find aPL at baseline in our patients with RA. Anti-cardiolipin autoantibodies were induced in 3.5% (2 of 57) and 1.8% (1 of 55) of our patients with RA, treated respectively with adalimumab or methotrexate only, whereas only one patient in the adalimumab group became positive for IgG and IgM anti-β_2_GPI autoantibodies. Moreover, the titers were all low and no clinical manifestations potentially related to the anti-phospholipid syndrome were recorded. Previous studies reported higher aPL frequencies both at baseline [44,45] and after anti-TNF-α therapy [8,9,16,19]. The difference with our results is probably related to the specificity of the assays we used, as demonstrated in a recent multicenter study [30].

Although at lower frequency than that reported with other TNF-α blocking agents, both ANA and aPL were clearly associated with adalimumab treatment. It has been suggested that a complex series of events related to TNF-α blockage might have a role in favoring the appearance of these autoantibodies. A dysregulation of apoptosis seems to be the most likely mechanism. Apoptotic cells do in fact expose nuclear antigens on their surface, and apoptotic blebs have been reported to expose anionic phospholipids (mainly phosphatidylserine) that in turn are able to bind circulating β_2_GPI; both phenomena might act as persistent immunogenic stimuli that could end in an antibody response against the self molecules [46,47]. In line with such a hypothesis is the recent demonstration that plasma levels of plasma nucleosomes were higher after infliximab treatment [48]. However, dysregulation and/or inhibition of the cytotoxic T lymphocyte response normally suppressing autoreactive B cells might be involved [17,49].

## Conclusion

Our results confirm the clinical efficacy of adalimumab, a new fully human anti-TNF-α monoclonal antibody, in improving the clinical score in patients with active RA that was not responsive to the conventional treatment. Such an effect is associated with a decrease in serum levels of RF and anti-CCP antibody levels, which was detected after 24 weeks and remained stable until 48 weeks of treatment. Induction of ANA, low-titer IgG/IgM anti-dsDNA but not aPL seems to be a drug-class-related effect, because increased antibody levels were found during treatment; however, no related clinical manifestations were recorded and antibody frequency was found to be lower than in studies with other TNF blocking agents.

## Abbreviations

aCL = anti-cardiolipin; ACR = American College of Rheumatology; ANA = antinuclear autoantibodies; aPL = anti-phospholipid autoantibodies; β_2_GPI = β_2 _glycoprotein I; CCP = cyclic citrullinated peptide; DAS 28 = Disease Activity Score; dsDNA = double-stranded DNA; ELISA = enzyme-linked immunosorbent assay; ENA = extractable nuclear antigens; ESR = erythrocyte sedimentation rate; RA = rheumatoid arthritis; RF = rheumatoid factor; TNF = tumor necrosis factor.

## Competing interests

The authors declare that they have no competing interests.

## Authors' contributions

FA and MC participated in the design of the study and in the clinical evaluation of the patients. DDA, GC and CC performed the immunoassays. PSP and PM participated in the design and coordination of the study and helped to draft the manuscript. SD participated in the study and performed the statistical analysis. All authors read and approved the final manuscript.

## Figures and Tables

**Table 1:** Clinical and demographic characteristics of the patients

Characteristic	Patients with RA	RA control group
Number of patients	57	55
Mean age, years (range)	56 (28–83)	63 (30–83)
Sex (F/M)	53/4	45/10
Disease duration, years (range)	8 (1–27)	6 (1–25)
Adalimumab treatment, *n*	57	0
Concomitant medications:		
NSAID	48	34
Corticosteroids	42	30
Methotrexate	57	55
Other	6	0

NSAID, non-steroidal anti-inflammatory drugs; RA, rheumatoid arthritis.

**Table 2:** Clinical characteristics of patients at baseline and after 24 and 48 weeks of adalimumab treatment

Variable	Week 0	Week 24	*P*	Week 48	*P*
DAS 28	5.4 ± 1.3	3.6 ± 1.2	<0.01	2.7 ± 0.9	<0.01
Tender joint count	12.4 ± 4.7	5.1 ± 3.5	<0.01	4.9 ± 3.5	<0.01
Swollen joint count	10.4 ± 3.8	3.2 ± 3.4	<0.01	3.12 ± 3.4	<0.01
ESR (mm/h)	35 ± 17	26 ± 16	<0.01	24 ± 15	<0.01
CRP (mg/dl)	42 ± 22.7	21 ± 15.2	<0.01	15 ± 14.8	<0.01
Anti-CCP (AU)^a^	116.9 ± 43.6	100.5 ± 46.5	<0.01	78.5 ± 43.9	<0.01
RF (IU)	121.7 ± 120.6	81 ± 90	<0.01	70.2 ± 82.7	<0.01

^a^Only anti-CCP positive patients at baseline (46 of 57) were included in the evaluation. Results are mean values ± SD. AU, arbitrary units; CCP, cyclic citrullinated peptide; CRP, C-reactive protein; DAS 28 = Disease Activity Score; ESR, erythrocyte sedimentation rate; RF, rheumatoid factor.

**Table 3:** Decrease of RF titers in adalimumab treated patients: correlation with the clinical response to treatment

ACR response, week 24	Week 0	Week 24	*P*	ACR response, week 48	Week 0	Week 48	*P*
<20% (*n *= 6)	130.5 ± 97.9	116.5 ± 88.6	n.s.	<20% (*n *= 3)	89 ± 73	60.3 ± 49.2	n.d.
ACR 20 (*n *= 22)	155.3 ± 147.5	109 ± 123	<0.0001	ACR 20 (*n *= 23)	164.5 ± 141.3	105.7 ± 112.6	<0.0001
ACR 50 (*n *= 21)	94.1 ± 60.9	57.3 ± 36.2	<0.0001	ACR 50 (*n *= 16)	96.4 ± 73.2	51.6 ± 41	0.0001
ACR 70 (*n *= 8)	94.8 ± 164.2	40.4 ± 54	n.s.	ACR 70 (*n *= 15)	89.5 ± 123.4	37.5 ± 40.7	0.018

Results are means ± SD. ACR, American College of Rheumatology; n.d., not done; n.s., not significant; RF, rheumatoid factor.

**Table 4:** Anti-CCP titer decrease in adalimumab treated patients: correlation with the clinical response to treatment

ACR response, week 24	Week 0	Week 24	*P*	ACR response, week 48	Week 0	Week 48	*P*
<20% (*n *= 6)	118.4 ± 34.9	111.8 ± 48.8	n.s.	<20% (*n *= 3)	107 ± 14.1	68.4 ± 23.3	n.d.
ACR 20 (*n *= 21)	111.5 ± 45.9	104.3 ± 48.5	n.s.	ACR 20 (*n *= 21)	121.8 ± 48.2	88.6 ± 52.6	0.001
ACR 50 (*n *= 16)	121.9 ± 45.1	93.9 ± 46.4	0.001	ACR 50 (*n *= 13)	119.6 ± 45.8	73.5 ± 36.2	0.001
ACR 70 (*n *= 3)	126.8 ± 51.9	85.9 ± 43.5	n.d.	ACR 70 (*n *= 9)	105.3 ± 37.7	65.3 ± 35.5	0.003

46 anti-CCP-positive patients at baseline were included in the evaluation. Results are means ± SD. CCP, cyclic citrullinated peptide; n.d., not done; n.s., not significant.

**Table 5:** Antinuclear antibody detection during adalimumab treatment

Treatment	Week	Number of positive ANA sera (%)		
		
		ANA	Anti-dsDNA	Anti-ENA
Adalimumab (*n *= 57)	0	4 (7)	0 (0)	3 (5.2)
	24	9 (16)	2 (3.5)	0
	48	12 (21)	4 (7)	2 (3.5)
Total		16 (28)	4 (7)	5 (9)
*P*		<0.01	<0.05	n.s.
Control RA (*n *= 55)	0	5 (9)	0	4 (7)
	24	2 (3.5)	0	0
	48	3 (5.2)	0	1 (1.7)
Total		8 (14.5)	0 (0)	5 (8.7)
*P*		n.s.		n.s.

ANA, antinuclear antibodies; anti-dsDNA, anti-double-stranded DNA autoantibodies; ENA, extractable nuclear antigens; n.s., not significant; RA, rheumatoid arthritis.

## References

[B1] Bathon JM, Martin RW, Fleischmann RM, Tesser JR, Schiff MH, Keystone EC, Genovese C, Wasko MC, Moreland LW, Weaver AW (2000). A comparison of etanercept and methotrexate in patients with early rheumatoid arthritis. N Engl J Med.

[B2] Elliott MJ, Maini RN, Feldmann M, Kalden JR, Antoni C, Smolen JS, Leeb B, Breedveld FC, Macfarlane JD, Bijl H (1994). Randomised double-blind comparison of chimeric monoclonal antibody to tumour necrosis factor alpha (cA2) versus placebo in rheumatoid arthritis. Lancet.

[B3] Lipsky PE, van der Heijde DM, St Clair EW, Furst DE, Breedveld FC, Kalden JR, Smolen JS, Weisman M, Emery P, Feldmann M (2000). Infliximab and methotrexate in the treatment of rheumatoid arthritis. Anti-Tumor Necrosis Factor Trial in Rheumatoid Arthritis with Concomitant Therapy Study Group. N Engl J Med.

[B4] Moreland L (2004). Adalimumab in rheumatoid arthritis. Curr Rheumatol Rep.

[B5] St Clair EW (2002). Infliximab treatment for rheumatic disease: clinical and radiological efficacy. Ann Rheum Dis.

[B6] Alessandri C, Bombardieri M, Papa N, Cinquini M, Magrini L, Tincani A, Valesini G (2004). Decrease of anti-cyclic citrullinated peptide antibodies and rheumatoid factor following anti-TNFα therapy (infliximab) in rheumatoid arthritis is associated with clinical improvement. Ann Rheum Dis.

[B7] Charles PJ, Smeenk RJ, De Jong J, Feldmann M, Maini RN (2000). Assessment of antibodies to double-stranded DNA induced in rheumatoid arthritis patients following treatment with infliximab, a monoclonal antibody to tumor necrosis factor-α: findings in open-label and randomised placebo-controlled trials. Arthritis Rheum.

[B8] Bobbio-Pallavicini F, Alpini C, Caporali R, Avalle S, Bugatti S, Montecucco C (2004). Autoantibody profile in rheumatoid arthritis during long-term infliximab treatment. Arthritis Res Ther.

[B9] Eriksson C, Engstrand S, Sundqvist KG, Rantapaa-Dahlqvist S (2005). Autoantibody formation in patients with rheumatoid arthritis treated with anti-TNFα. Ann Rheum Dis.

[B10] De Rycke L, Verhelst X, Kruithof E, Van den Bosh F, Hoffman IE, Veys EM, De Keyser F (2005). Rheumatoid factor, but not anti-cyclic citrullinated peptide antibodies, is modulated by infliximab treatment in rheumatoid arthritis. Ann Rheum Dis.

[B11] Caramaschi P, Biasi D, Tonolli E, Pieropan S, Martinelli N, Carletto A, Volpe A, Bambara LM (2005). Antibodies against cyclic citrullinated peptides in patients affected by rheumatoid arthritis before and after infliximab treatment. Rheumatol Int.

[B12] Antivalle M, Marazza MG, Randisi G, Sarzi-Puttini P, Dell'Acqua D, Carrabba M (2002). Longterm treatment of rheumatoid arthritis with infliximab: efficacy, side effects and autoantibodies induction [abstract]. Arthritis Rheum.

[B13] De Rycke L, Kruithof E, Van Damme N, Hoffman IE, Van den Bossche N, Van den Bosch F, Veys EM, De Keyser F (2003). Antinuclear antibodies following infliximab treatment in patients with rheumatoid arthritis or spondylarthropathy. Arthritis Rheum.

[B14] Louis M, Rauch J, Armstrong M, Fitzcharles MA (2003). Induction of autoantibodies during prolonged treatment with infliximab. J Rheumatol.

[B15] Allanore Y, Sellam J, Batteux F, Job Deslandre C, Weill B, Kahan A (2004). Induction of autoantibodies in refractory rheumatoid arthritis treated by infliximab. Clin Exp Rheumatol.

[B16] Ferraro-Peyret C, Coury F, Tebib JG, Bienvenu J, Fabien N (2004). Infliximab therapy in rheumatoid arthritis and ankylosing spondylitis-induced specific antinuclear and antiphospholipid autoantibodies without autoimmune clinical manifestations: a two-year prospective study. Arthritis Res Ther.

[B17] Ziolkowska M, Maslinski W (2003). Laboratory changes on anti-tumor necrosis factor treatment in rheumatoid arthritis. Curr Opin Rheumatol.

[B18] Debandt M, Vittecoq O, Descamps V, Le Loet X, Meyer O (2003). Anti-TNFα-induced systemic lupus erythematosus. Clin Rheumatol.

[B19] Ferraccioli G, Mecchia F, Di Poi E, Fabris M (2002). Anticardiolipin antibodies in rheumatoid patients treated with etanercept or conventional comnination therapy: direct and indirect evidence for a possible association with infections. Ann Rheum Dis.

[B20] Jonsdottir T, Forslid J, van Vollenhoven A, Harju A, Brannenmark S, Klareskog L, van Vollenhoven RF (2004). Treatment with anti-TNFα antagonists in patients with rheumatoid arthritis induces anticardiolipin antibodies. Ann Rheum Dis.

[B21] Weinblatt ME, Keystone EC, Furst DE, Moreland LW, Weisman MH, Birbara CA, Teoh LA, Fischkoff SA, Chartash EK (2003). Adalimumab, a fully human anti-tumor necrosis factor alpha monoclonal antibody, for the treatment of rheumatoid arthritis in patients taking concomitant methotrexate: the ARMADA trial. Arthritis Rheum.

[B22] Burmester GR, Monteagudo Saez I, Malaise M, Canas da Silva J, Webber DG, Kupper H (2004). Efficacy and safety of Adalimumab (HUMIRA) in European Clinical Practice: The ReAct trial. Ann Rheum Dis.

[B23] Arnett FC, Edworthy SM, Bloch DA, McShane DJ, Fries JF, Cooper NS, Healey LA, Kaplan SR, Liang MH, Luthra HS (1988). The American Rheumatism Association 1987 revised criteria for the classification of rheumatoid arthritis. Arthritis Rheum.

[B24] van der Heijde DM, van't Hof M, van Riel PL, van de Putte LB (1993). Development of a disease activity score based on judgment in clinical practice by rheumatologists. J Rheumatol.

[B25] Felson DT, Anderson JJ, Boers M, Bombardier C, Furst D, Goldsmith C, Katz LM, Lightfoot R, Paulus H, Strand V (1995). American College of Rheumatology. Preliminary definition of improvement in rheumatoid arthritis. Arthritis Rheum.

[B26] Avcin T, Cimaz R, Falcini F, Zulian F, Martini G, Simonini G, Porenta-Besic V, Cecchini G, Borghi MO, Meroni PL (2002). Prevalence and clinical significance of anti-cyclic citrullinated peptide antibodies in juvenile idiopathic arthritis. Ann Rheum Dis.

[B27] Bayer PM, Bauerfeind S, Bienvenu J, Fabien N, Frei PC, Gilburd B, Heide KG, Hoier-Madsen M, Meroni PL, Monier JC (1999). Multicenter evaluation study on a new HEp2 ANA screening enzyme immune assay. J Autoimmun.

[B28] Fritzler MJ, Conrad K, Bachmann MP, Chan EKL, Fritzler MJ, Humbel RL, Sack U, Shoenfeld Y (2004). New technologies in the detection of autoantibodies evaluation of addressable laser bead immunoassays (ALBIA). Autoantigens, Autoantibodies, Autoimmunity.

[B29] Riboldi P, Gerosa M, Moroni G, Radice A, Sinico A, Tincani A, Meroni PL (2005). Anti-DNA antibodies: a diagnostic and prognostic tool for systemic lupus erythematosus?. Autoimmunity.

[B30] Tincani A, Allegri F, Sanmarco M, Cinquini M, Taglietti M, Balestrieri G, Koike T, Ichikawa K, Meroni P, Boffa MC (2001). Anticardiolipin antibody assay: a methodological analysis for a better consensus in routine determinations – a cooperative project of the European Antiphospholipid Forum. Thromb Haemost.

[B31] Bukhari M, Lunt M, Harrison BJ, Scott DG, Symmons DP, Silman AJ (2002). Rheumatoid factor is the major predictor of increasing severity of radiographic erosions in rheumatoid arthritis: results from the Norfolk Arthritis Register Study, a large inception cohort. Arthritis Rheum.

[B32] Schellekens GA, Visser H, de Jong BA, van den Hoogen FH, Hazes JM, Breedveld FC, van Venrooij WJ (2000). The diagnostic properties of rheumatoid arthritis antibodies recognizing a cyclic citrullinated peptide. Arthritis Rheum.

[B33] Kroot EJ, de Jong BA, van Leeuwen MA, Swinkels H, van den Hoogen FH, van't Hof M, van de Putte LB, van Rijswijk MH, van Venrooij WJ, van Riel PL (2000). The prognostic value of anti-cyclic citrullinated peptide antibodies in patients with recent-onset rheumatoid arthritis. Arthritis Rheum.

[B34] Meyer O, Labarre C, Dougados M, Goupilee P, Cantagrel A, Dubois A, Nicaise-Roland P, Sibilia J, Combe B (2003). Anti-citrullinated protein/peptide antibody assay in early rheumatoid arthritis for predicting five year radiographic damage. Ann Rheum Dis.

[B35] Bas S, Genevay S, Metyer O, Gabay C (2003). Anti-cyclic citrullinated peptide antibodies, IgM and IgA rheumatoid factors in the diagnosis and prognosis of rheumatoid arthritis. Rheumatology (Oxford).

[B36] van Venrooij WJ, Hazes JM, Visser H (2002). Anticitrullinated protein/peptide antibody and its role in the diagnosis and prognosis of early rheumatoid arthritis. Neth J Med.

[B37] Chen HA, Lin KC, Chen CH, Liao HT, Wang HP, Chang HN, Tsai CY, Chou CT The effect of etanercept on anti-cyclic citrullinated peptide antibodies and rheumatoid factor in patients with rheumatoid arthritis. Ann Rheum Dis.

[B38] Yazdani-Biuki B, Standlmainer E, Mulabecirovic A, Brezinschek R, Tilz G, Demel U, Mueller T, Brickmann K, Graninger WB, Brezinschek HP (2005). Blockade of tumor necrosis factor α significantly alters the serum levels of IgG- and IgA-rheumatoid factor in patients with rheumatoid arthritis. Ann Rheum Dis.

[B39] Mikuls TR, O'Dell JR, Stoner JA, Parrish LA, Arend WP, Norris JM, Holers VM (2004). Association of rheumatoid arthritis treatment response and disease duration with declines in serum levels of IgM rheumatoid factor and anti-cyclic citrullinated peptide antibody. Arthritis Rheum.

[B40] Furst DE (1990). Rational use of disease-modifying antirheumatic drugs. Drugs.

[B41] Inagaki M, Takahara H, Nishi Y, Sugawara K, Sato C (1989). Ca^2+^-dependent deimination induced disassembly of intermediate filaments involves specific modification of the amino-terminal head domain. J Biol Chem.

[B42] Lugering A, Schmidt M, Lugering N, Pauels HG, Domaschke W, Kucharzik T (2001). Infliximab induces apoptosis in monocytes from patients with chronic active Crohn's disease by using a caspase-dependent pathway. Gastroenterology.

[B43] Keystone E, Haraoui B (2004). Adalimumab therapy in rheumatoid arthritis. Rheum Dis Clin North Am.

[B44] Keane A, Woods R, Dowding V, Roden D, Barry C (1987). Anticardiolipin antibodies in rheumatoid arthritis. Br J Rheumatol.

[B45] Wolf P, Gretler J, Aglas F, Auer-Grumbach P, Rainer F (1994). Anticardiolipin antibodies in rheumatoid arthritis: their relation to rheumatoid nodules and cutaneous vascular manifestations. Br J Dermatol.

[B46] Casciola-Rosen L, Rosen A, Petri M, Schlissel M (1996). Surface blebs on apoptotic cells are sites of enhanced procoagulant activity: implications for coagulation events and antigenic spread in systemic lupus erythematosus. Proc Natl Acad Sci USA.

[B47] Price BE, Rauch J, Shia MA, Walsh MT, Lieberthal W, Gilligan HM, O'Laughlin T, Koh JS, Levine JS (1996). Anti-phospholipid autoantibodies bind to apoptotic, but not viable, thymocytes in a beta 2-glycoprotein I-dependent manner. J Immunol.

[B48] D'Auria F, Rovere-Querini P, Giazzon M, Ajello P, Baldissera E, Manfredi AA, Sabbadini MG (2004). Accumulation of plasma nucleosomes upon treatment with anti-tumour necrosis factor-alpha antibodies. J Intern Med.

[B49] Via CS, Shustov A, Rus V, Lang T, Nguyen P, Finkelman FD (2001). *In vivo* neutralization of TNF-alpha promotes humoral autoimmunity by preventing the induction of CTL. J Immunol.

